# Impact of anthropogenic pressures on wild mammals of Northern Portugal

**DOI:** 10.14202/vetworld.2020.2691-2702

**Published:** 2020-12-18

**Authors:** Andreia Garcês, Isabel Pires, Fernando Pacheco, Luís Sanches Fernandes, Vanessa Soeiro, Sara Lóio, Justina Prada, Rui Cortes, Felisbina Queiroga

**Affiliations:** 1 Centre for the Research and Technology of Agro-Environment and Biological Sciences, University of Trás-os-Montes and Alto Douro, Vila Real, Portugal; 2 Department of Veterinary Science, University of Trás-os-Montes and Alto Douro, Vila Real, Portugal; 3 CECAV, University of Trás-os-Montes and Alto Douro, Vila Real, Portugal; 4 Chemistry Research Centre, University of Trás-os-Montes and Alto Douro, Vila Real, Portugal; 5 Wildlife Rehabilitation Centre of Parque Biológico de Gaia, R. Cunha, Avintes, Portugal

**Keywords:** anthropogenic factors, mortality, Northern of Portugal, partial least squares path modeling, wild mammals

## Abstract

**Background and Aim:**

Wild mammals are among the most threatened species of the world in large part due to human activity. In this work, we used the method of partial least squares-path modeling associated with a geographic information system to analyze the impact of anthropogenic pressures on the mortality of wild mammals.

**Materials and Methods:**

We collected the data related to the cause of death of native wild mammals admitted to the Wildlife Rehabilitation Centre of Parque Biológico de Gaia in Northern Portugal, during 10 years (2008-2017).

**Results:**

A total of 359 animals from 42 municipalities (rural and urban areas) were included in the study. The main cause of death was of traumatic origin. From the anthropogenic pressures included in the study, water reservoirs, small companies, and residential buildings were the ones that contributed the most to increase the mortality of traumatic and non-traumatic origin. This relation of cause-effect (mortality-anthropogenic pressures) was supported by the high coefficients of determination obtained (R^2^ > 0.8).

**Conclusion:**

The present results allow a general view on the reality of mammal’s mortality in Northern Portugal. Furthermore, it could also constitute a valuable tool for the conservation of wild mammals in those areas.

## Introduction

Many wild mammal populations are declining around the globe, following the similar trends of other species. At present, more than 34,000 species are categorized as threatened worldwide [1]. Every year numerous wild mammals die as a consequence of anthropogenic hazards, such as collision with vehicles, poisoning, poaching, pet/part of animals trade, habitat destruction, and predation by domestic pets [2-4]. The search for statistical associations between anthropogenic factors and causes of mortality helps to identify the factors that are harmful to wildlife populations. The conclusions taken from that research can provide holistic views on cause-effect relationships that are essential for the establishment of preventive measures related to the conservation of wild animals and their habitat [5]. So far, some studies have been conducted worldwide on the impact of anthropogenic pressures on wild mammal populations, focused on specific species such as European mink (*Mustela lutreola*) [6], bats [7], bobcats (*Lynx rufus*), coyotes (*Canis* *latrans*) [8], and Florida black bears (*Ursus americanus floridanus*) [9].

In Northern Portugal, studies focused on understanding the anthropogenic impact of human activity in wild animals are scarce. Our team published a very recent study concerning the impact of anthropogenic stressors in wild birds [10], but no information is available for wild mammals. There are some studies published on bats in the United Kingdom [11] and brown bear (*Ursus arctos*) [12] in Slovakia. In both cases, the researchers investigated the link between mortality and the existence of roads in a given spatial area. The present work moves forward and not only analyses the influence of roads in wild mammals’ mortality but also other human pressures such as the density of human population, buildings, burnt areas or farms, and among others. To accomplish the purpose, the authors applied the method of partial least squares-path modeling (PLS-PM) in this study. This model was selected because of its minimum demand on the sample size and the high levels of statistical significance that can achieve [13]. The PLS-PM has been used by researchers from different areas such as social studies or ecology [14-16]. The PLS-PM method is based on structural equations composed of multiple regressions (the measurement or outer model) and principal components (the structural or inner model), which allow estimating complex cause-effect relationships among latent variables [15-17]. It is important to refer that the North of Portugal is characterized by a Mediterranean climate with mild temperatures, plateaus, and mountains (where predominate Atlantic vegetation) and by the presence of an extensive maritime line. It is also one of the most industrialized regions of the country, based on small companies [18] mostly related to commerce (20%), agriculture and livestock production (8%), restoration (7%), and construction (7%) [19].

To the authors’ best knowledge, the use of PLS-PM in environmental assessments involving wild mammals has not been tempted so far. The purpose of this work was, therefore, to use the PLS-PM method to investigate the impact of diverse anthropogenic pressures on the mortality of wild mammals admitted to the Wildlife Rehabilitation Centre of Parque Biológico de Gaia from 2008 to 2017.

## Materials and Methods

### Ethical approval

This study received the approval of the Department of School of Veterinary Medicine from UTAD and was performed in compliance with the Portuguese legislation for the protection of animals (Law nº 92/1995, from September the 12^th^)

### Study population and spatial area

**Figure-1:**
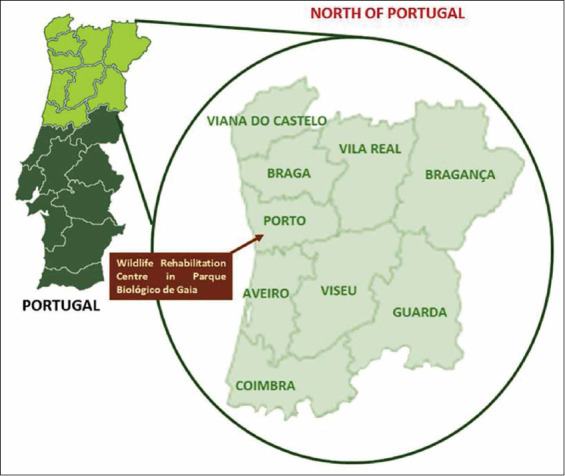
Map with the representation of Northern Portugal, highlighting the nine districts included in the study.

The medical records of wild mammals admitted to the Wildlife Rehabilitation Centre (WRC) (41° 05’ 48.50”N-8° 33’ 21.34”W) from 2008 to 2017 were analyzed. Animals were collected from the nine districts of Northern Portugal (Figure-1).

### Sample selection and definition of variables for PLS-PM

The admission criteria followed for this study were the inclusion of animals that died during admission or treatment at the WRC or that were euthanized in accordance to their unfavorable prognosis for subsequent release back into the natural habitat.

Individual data were categorized according to the mammal taxonomic order, species, and age (adult >1-year and juvenile < 1-year). Since information related to sex was absent in the majority of the animals’ admittance records, this characteristic was not considered for the study.

The inventory of death causes is in keeping with a previous publication [10]. Non-traumatic death causes comprise nutritional disorders, parasitism, poisoning, infectious diseases, and non-trauma of unknown origin. Traumatic causes include predation, collision with vehicles, collision with buildings, and trauma of unknown origin.

### Statistical analysis

The descriptive statistics were performed using the Statistical Package for the Social Sciences (https://www.ibm.com/analytics/spss-statistics-software)version 24, advanced Models TM 21.0 (SPSS Inc., Chicago, IL 60606-6412, USA). The XLStat software (https://www.xlstat.com/en/) (Addinsoft, Paris, France) was used to prepare the PLS-PM dataset, to implement the structural equation model and save the results. The ArcMap software (http://desktop.arcgis.com/en/arcmap/) was used to handle spatial data and produce thematic maps. These computer packages are widely used in environmental studies [20-31].

The PLS-PM model was run twice with the purpose to investigate the non-traumatic and traumatic mortality in separate. It was structured as a formative model to handle the measured variables as causes of the latent variables “anthropogenic pressures” and “mortality.” The causes of mortality were defined in section 2.2 and the selected anthropogenic pressures were: Human population density, annual precipitation, number of domestic landfills, burnt area, number of water reservoirs, wind farms, roads, residential and non-residential buildings, number of farms, number of small companies (with <10 workers), medium companies (with 10-250 workers), and large companies (with >250 workers).

The data were compiled in a worksheet comprising n rows and p columns and representing the sample size and characterization, respectively. The sample size designates the number of municipalities from Northern Portugal where the animals were collected. The sample characterization encompassed the number of measured parameters, which includes the causes of mortality and the anthropogenic pressures. Although the causes of mortality can be very different among species, the PLS-PM model treated all species equally because consideration of species individually would in some cases reduce the sample size to a very small n hampering any statistical approach to the database.

The links between the two latent variables were quantified through path coefficients and the links between latent variables and measurable variables were quantified through weights. The coefficient of determination (R^2^) was used to represent the inner model’s goodness of fit. The path coefficients accounted for the influence of the pressures on mortality. The weights represent the contribution of each measured variable to its latent variable.

In the maps, the classes were set up based on sample population quantiles. In all cases, the terms “very high,” “high,” and so forth were used to distinguish classes of larger or lower values and capture their spatial incidences; they were not meant to ascribe absolute importance to the pressure or mortality cause.

## Results

### Study population

In total, 359 wild mammals belonging to seven different orders were included in the present study as follows: Order Eulipotyphla (n=163, 45.4%), Order Carnivora (n=81, 22.7%), Order Chiroptera (n=63, 17.5%), Order Lagomorpha (n=21, 5.8%), Order Artiodactyla (n=15, 4.2%), Order Rodentia (n=15, 4.2%), and Order Cetacea (n=1, 0.3%).

Regarding the age, 303 were adults (84.4%) and 56 (15.6%) were juveniles. The majority was admitted during spring (n=101, 28.10%) or summer (n=122, 34%), followed by autumn (n=95, 26.5%) and winter (n=41, 11.4%). Additional information for each species is available in Table-1. The animals were collected from 42 municipalities. The municipalities with the highest number of events were Vila Nova de Gaia (n= 137), Porto (n= 32), and Matosinhos (n=17).

**Table-1:** Frequency of admission in the Wildlife Rehabilitation Centre of Parque Biológico de Gaia and demographic data and conservation status of the wild mammals included in the study (2008-2017).

Species descriptive	Number of cases	Age		Conservation status
	
	n (%)	Adult	Juvenile	
Order Artiodactyla				
*Capreolus capreolus*	7 (1.9)	7 (1.9)	0 (0.0)	LC
*Sus scrofa*	6 (1.7)	6 (1.7)	0 (0.0)	LC
*Cervus elaphus*	1 (0.3)	1 (0.3)	0 (0.0)	LC
*Dama dama*	1 (0.3)	1 (0.3)	0 (0.0)	LC
Order Carnivora				
*Vulpes vulpes*	46 (12.8)	39 (10.9)	7 (1.9)	LC
*Mustela vison*	2 (0.6)	2 (0.6)	0 (0.0)	LC
*Martes foina*	6 (1.7)	4 (1.1)	2 (0.6)	LC
*Genetta genetta*	10 (2.8)	9 (2.5)	1 (0.3)	LC
*Neovison vison*	2 (0.6)	0 (0.0)	2 (0.6)	LC
*Meles meles*	2 (0.6)	1 (0.3)	1 (0.3)	LC
*Herpestes ichneumon*	1 (0.3)	0 (0.0)	1 (0.3)	LC
*Martes martes*	2 (0.6)	1 ((0.3)	1 (0.3)	DD
*Mustela putories*	2 (0.6)	2 (0.6)	0 (0.0)	DD
*Lutra lutra*	8 (2.2)	5 (1.4)	3 (0.8)	LC
Order Cetacea				
*Phocoena phocoena*	1 (0.3)	1 (0.3)	0 (0.0)	LC
Order Chiroptera				
*Pipistrelus pipistrelus*	60 (16.7)	57 (15.9)	3 (0.8)	LC
*Eptesicus serotinus*	1 (0.3)	1 (0.3)	0 (0.0)	LC
*Plecotus auritus*	1 (0.3)	0(0.0)	1 (0.3)	DD
*Myotis myotis*	1 (0.3)	0 (0.0)	1 (0.3)	LC
Order Eulipotyphla				
*Erinaceus europaeus*	159 (44.3)	127 (35.4)	32 (8.9)	LC
*Talpa europaea*	4 (1.1)	4 (1.1)	0 (0.0)	LC
Order Lagomorpha				
*Oryctolagus cuniculus*	21 (5.8)	21 (5.8)	0 (0.0)	NT
Order Rodentia				
*Sciurus vulgaris*	12 (3.3)	11 (3.1)	1 (0.3)	LC
*Apodemus sylvaticus*	3 (0.8)	2 (0.6)	1 (0.3)	LC

CR=Critically in danger, EN=Endangered, DD=Insufficient information, VU=Vulnerable, NT=Near threatened

### Wild mammals’ mortality

Concerning the cause of death, 127 cases (35.4%) were related to the non-traumatic origin and the remaining 232 cases (64.6%) to traumatic origin (Table-2). Most of the animals were diagnosed with a cause of death from unknown etiology (68 for non-trauma and 151 for trauma). The animals with a definitive diagnosis of the non-traumatic cause of death were in their majority due to nutritional disorders (n=44), with particular emphasis on the Order Carnivora (n=13) and Order Eulipotyphla (n=15). These animals were mostly orphans admitted to the WRC. Some of the infectious diseases (n=7) that were detected were leptospirosis (n=1) in the Order Carnivora; enteritis of unknown origin in Order Rodentia (n=1) and Order Artiodactyla (n=1); osteomyelitis (n=1) in Order Eulipotyphla; and myxomatosis (n=1) and rabbit hemorrhagic disease (n=1) in Order Lagomorpha. The parasitic diseases (n=7) were mostly related to a high prevalence of ectoparasites in the Order Eulipotyphla (n=2) and Lagomorpha (n=2). Only one animal, a hedgehog (*Erinaceus europaeus*) presented signals compatible with poisoning. Concerning the traumatic causes of death, 56 animals died due to collision with vehicles, with particular emphases on the Order Carnivora (n=14) and Order Eulipotyphla (n=31). Two animals from the Order Eulipotyphla and Order Carnivora died due to gunshot. Predation occurred mostly in Order Eulipotyphla (n=8), Order Carnivora (n=4), and Order Chiroptera (n=3). Collision with buildings affected mostly animals from Order Chiroptera (n=5) and Order Rodentia (n=3).

**Table-2:** Mortality causes by different orders of mammals admitted to the Wildlife Rehabilitation Centre from 2008 to 2017.

Orders	Trauma: Number of cases (%)					Non-trauma: Number of cases (%)				
	
	Collision vehicles	Collision buildings	Predation	Gunshot	Unknown trauma	Toxic	Infectious diseases	Nutritional disorders	Parasitic diseases	Unknow non-traumatic
Artiodactyla	2 (0.6)	0 (0.0)	0 (0.0)	0 (0.0)	11 (3.10)	0 (0.0)	1 (0.3)	0 (0.0)	0 (0.0)	1 (0.3)
Carnivora	14 (3.9)	1 (0.3)	4 (1.1)	1 (0.3)	36 (10.0)	0 (0.0)	1 (0.3)	13 (3.6)	1 (0.3)	10 (2.8)
Cetacea	0 (0.0)	0 (0.0)	0 (0.0)	0 (0.0)	0 (0.0)	0 (0.0)	0 (0.0)	0 (0.0)	1 (0.3)	0 (0.0)
Chiroptera	1 (0.3)	5 (1.4)	3 (0.8)	0 (0.0)	36 (10.0)	0 (0.0)	1 (0.3)	3 (0.8)	1 (0.3)	13 (3.6)
Eulipotyphla	31 (8.6)	0 (0.0)	8 (2.2)	1 (0.3)	60 (16.7)	1 (0.3)	1 (0.3)	25 (7.0)	2 (0.6)	31 (8.6)
Lagomorpha	3 (0.8)	1 (0.3)	1 (0.3)	0 (0.0)	5 (1.4)	0 (0.0)	2 (0.6)	0 (0.0)	2 (0.6)	7 (1.9)
Rodentia	1 (0.3)	3 (0.8)	1 (0.3)	0 (0.0)	3 (0.8)	0 (0.0)	1 (0.3)	3 (0.8)	0 (0.0)	6 (1.7)

When looking at the specific cause non-traumatic causes of mortality, the municipalities ranked in the top position are: (a) Nutritional disorders – Vila Nova de Gaia (n=5); (b) parasitism – Vila Nova de Gaia (n=4); (c) poisoning – Santo Tirso (n=1); (d) infectious diseases – Vila Nova de Gaia (n=3); and (e) unknown non-trauma – Vila Nova de Gaia (n=41).

When looking at the specific cause of traumatic death, the municipality ranked in the top position was Vila Nova de Gaia: (a) Collision with vehicles (n=12); (b) predation (n=7); (c) collision with building (n=6); and (d) unknown trauma (n=59).

### Anthropogenic pressures and wild mammal’s mortality

#### Anthropogenic pressures

**Figure-2:**
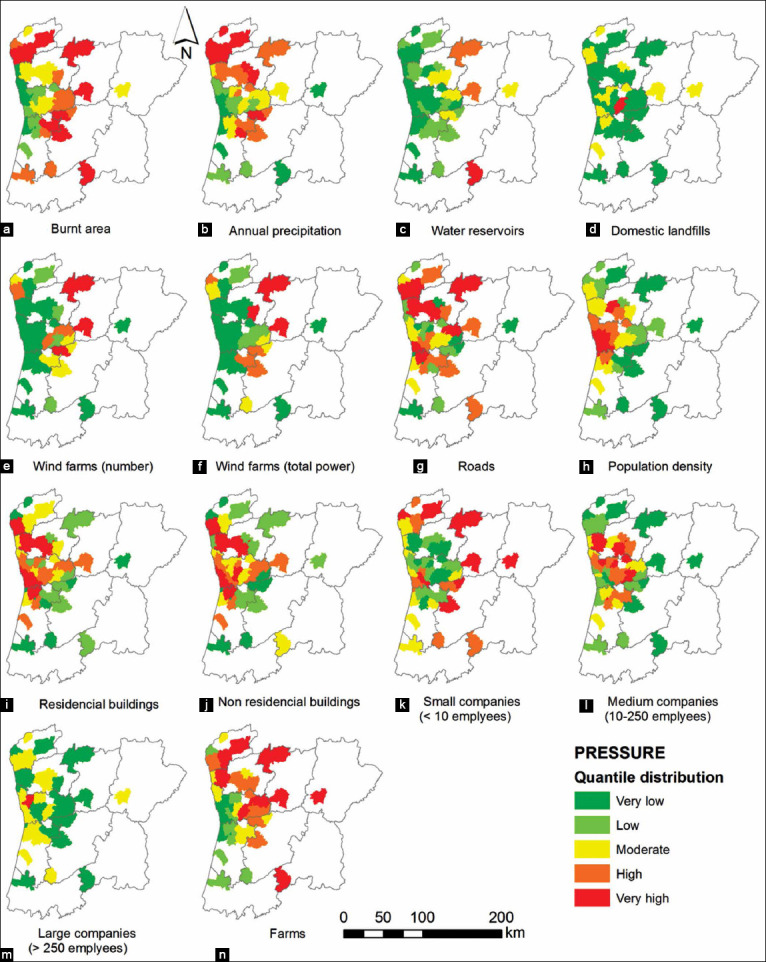
(a-n) Spatial distribution of specific anthropogenic pressures by the different municipalities in the North of Portugal (2008-2017).

The anthropogenic pressures roads, population density, residential and non-residential buildings, and medium companies, increase from the inland to the coastal areas. For the remaining parameters, the results reflect an asymmetry between the rural inland and the urban coast. The factors burnt areas, annual precipitation, water reservoirs, landfills, small and large companies and farms are predominant in the inland areas. The wind farms predominate in the central area since various mountain ridges are situated in this region, which allows the installation of effective wind speeds. Finally, the factor roads are more or less uniform across the studied municipalities, which probably relates to the fact that roadways are viewed as a public infrastructure at the service of territorial cohesion (Figure-2).

#### Wild mammal’s mortality

**Figure-3:**
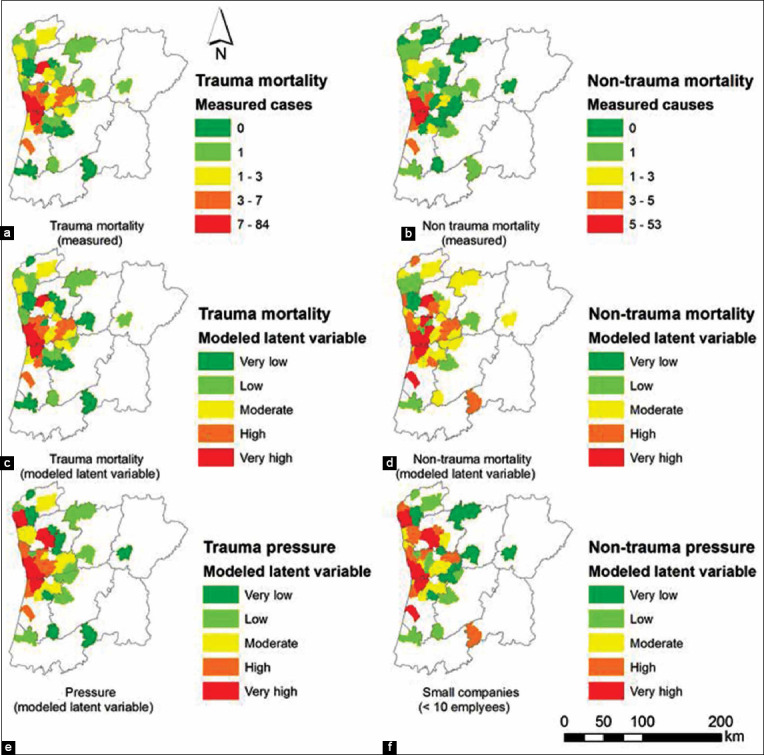
(a-f) Comparative spatial distribution of measured and modeled total trauma and non-trauma mortality model in the wild mammals admitted to the Wildlife Rehabilitation Centre of Parque Biológico de Gaia from municipalities of Northern Portugal (2008-2017).

The spatial distribution of trauma and non-trauma mortality based on the measured number of cases (Figure-3a and b) as well as based on the results of PLS-PM (Figure-3c and d) is fully described in Figure-3. The correspondence between the measured and modeled distributions is noticeable, which favors the applicability of PLS-PM in this study.

**Figures-4:**
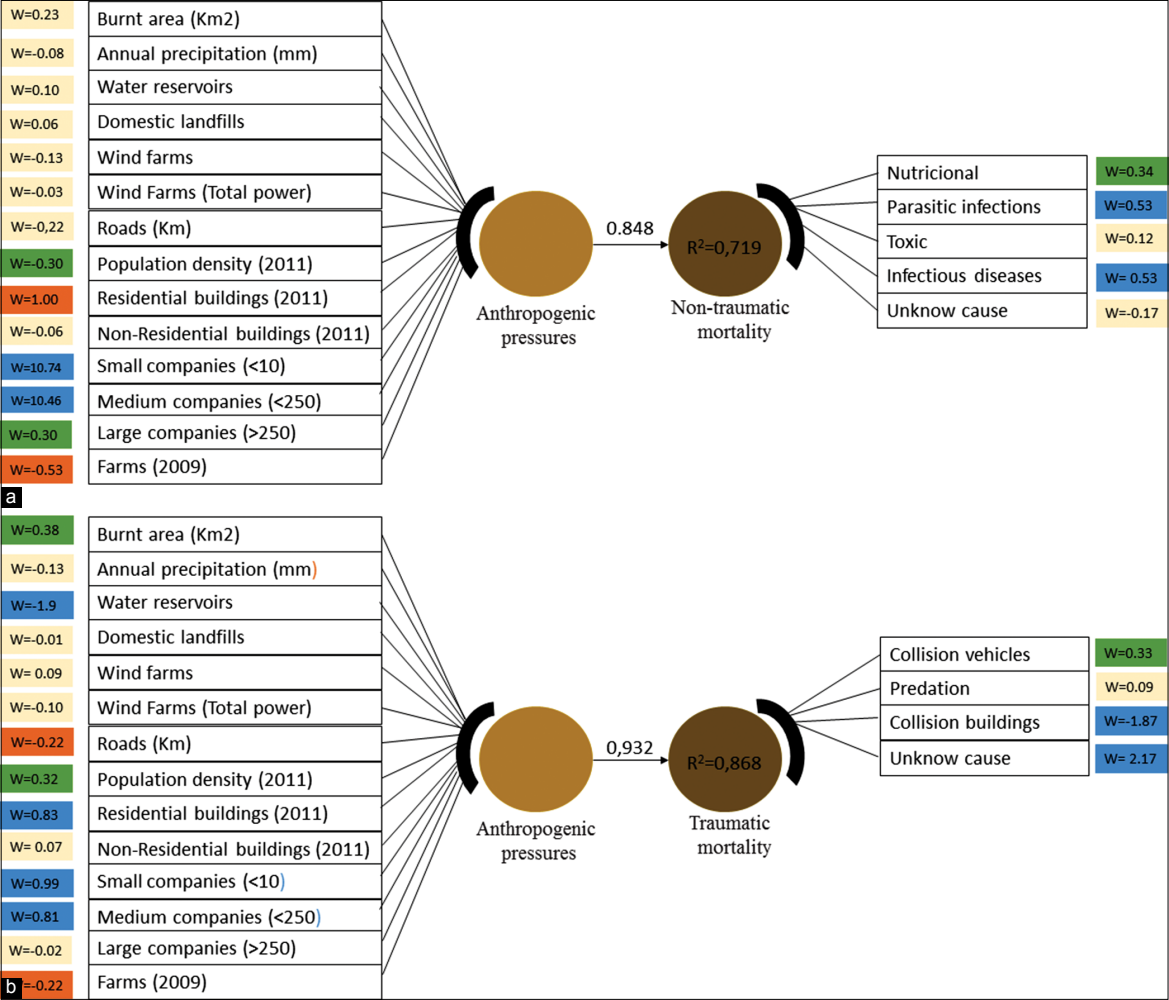
(a and b) Diagram illustrating the anthropogenic pressures and traumatic/non-traumatic causes of mortality in wild mammals admitted to the Wildlife Rehabilitation Centre from 2008 to 2017. Panel A refers to the non-traumatic model and panel B to traumatic model. The colored rectangles represent the measurable variables, the colored circles the corresponding anthropogenic pressures. The arrows represent the link between latent variables. The blue colored rectangles represent the death causes and anthropogenic pressures with a bigger impact on the wild mammal’s mortality, followed by the green and orange colored rectangles. The rectangles shaded as yellow have quite a small impact.

The results obtained with PLS-PM demonstrate the relationship between anthropogenic pressures and non-traumatic/traumatic causes of mortality (Figure-4a and b). The corresponding path coefficients are 0.848 (with R^2^=0.72) for the non-trauma case and 0.932 (with R^2^=0.87) for the trauma-case.

The PLS-PM model of non-traumatic mortality (Figure-4a) identified the mortality associated with parasitic and infectious diseases as the main causes of death associated with the anthropogenic pressures under study. According to the results, the main anthropogenic pressures related to wild mammal’s non-traumatic mortality were small (w=10.74) and medium companies (w=10.46). In the second line of influence, the pressures residential buildings concentration (w=–1.00) and farms density (w=–0.53) were identified. Some other pressures were less important considering their smaller absolute weights, as large companies (w=0.30) and population density (w=–0.30). Burnt areas, annual precipitation, water reservoirs, domestic landfills, wind farms, roads, and non-residential buildings had insignificant or no impact on the mortality of non-traumatic causes.

The PLS-PM model of traumatic mortality (Figure-4b) identified the mortality due to collision with buildings and trauma of unknown origin as the main causes of death associated with the anthropogenic pressures under study. The main anthropogenic pressures identified were water reservoirs (w=–1.9), small companies (w=0.99), medium companies (w=0.81), and residential buildings (w=0.83). Roads (w=–0.2), farms (w=–0.22), population density (w=0.32), and burnt areas (w=0.38) have also impact on mortality, although with less relevance. The remaining pressures had insignificant or no impact on mortality.

It is possible to conclude based on our data that human pressures have a bigger impact on non-traumatic mortality. This is because the larger weights (10.74; 10.46) in the most relevant pressures (water reservoirs and small and medium companies) are represented in the non-traumatic mortality model. Our interpretation of these results is reinforced by the fact that both models were based on the same sample and anthropogenic pressures, even though the magnitude of the weights is dependent on numerous conditions [32].

## Discussion

Wild mammals represent the second most abundant class of vertebrates in Northern Portugal (after birds), with a total of 87 terrestrial and marine species [33]. Due to its privileged location in a transition zone between two major climatic and biogeographic regions (the Atlantic and the Mediterranean), Portugal constitutes a refuge for a great variety of species with diverse environmental affinities. The northern region of Portugal has the richest populations of mammal species, along with regions Alentejo and Algarve [34].

In the present study, among the 359 animals admitted to the WRC, the main cause of death was due to traumatic origin (64.6%). This is similar to observations by other researchers [8,12,35-37]. Our data indicate that trauma mortality was mainly due to unknown origin, followed by predation and collision with buildings. The elevated percentage of animals that died of the trauma of unknown origin could be due to the lack of information in the medical records but also to the financial and/or logistic impossibility to perform complementary diagnostic exams. Necropsy is not always done due to many reasons such as lack of time to perform the exam and few technicians or improper installations and material, which impairs getting a definitive diagnosis concerning the cause of death [38-40].

Our data also revealed that areas with the highest density of dead animals that were admitted to the WRC are located near the coast, particularly in urban areas such as Vila Nova de Gaia and Porto. In the maps of Figure-2, it is possible to observe that the majority of the selected anthropogenic pressures are also located in those areas, facilitating the interaction of human-animals and, therefore, its negative effects [10]. The higher concentration of prey, the increase in the success of catches and the use of man-made structures for shelter and reproduction attract some mammal species (e.g., foxes, bats, and hedgehogs) to adopt urban biomes as their new habitat [41], which can also be a justification for the elevated number of wild mammals found dead in urban areas. Moreover, as expected, most of the animals were collected from areas near the location of the WRC. This can be related to the fact that the population of these areas is aware of the existence of the center and bring the injured animals there when they find one. This factor also can imply a significant deviation of the data.

The present manuscript establishes a correlation between the mortality of wild mammals admitted to the WRC and potentially threatening anthropogenic pressures using PLS-PM while illustrating the spatial incidence of both groups of variables. The study lasted for an extensive period of time (10 years). To the authors’ best knowledge, there are no similar reports on the literature involving wild mammals. This model has been used before and demonstrated that human activity may have a great impact on the loss of biodiversity [10]. It is worth to mention that weights are positive for most measured variables and negative for a few others. The presence of negative signs might be related to the “reverse coding” of regionalized variables. In the PLS-PM context, negative signs can be linked to many motives, as the presence of unlikely causal relationships [42] or the variability caused by collinearity [43] - Simpson’s paradox. For example, the number of water reservoirs or farms is variables that increase toward the inland area (Figure-2c and n) whereas population density (Figure-2h) or the number of medium companies (Figure-2l) increases in the opposite direction. As an example, in the model of traumatic mortality (Figure-2b), the negative spatial correlation between farms or water reservoirs and the other two variables would lead to the same weight signs of population density and number of medium companies (+0.32; +0.81) and different weight signs in water reservoirs or farms (–1,9; –0.22). This would occur unrelatedly to the impact of these four anthropogenic pressures on traumatic mammals’ mortality. Another justification for the negative signs can be related to reversing coding of regionalized variables. An example, some variables such as roads (–0.02 traumatic mortality model; and –0.22 non-traumatic mortality model, Figure-2g) do not display any obvious spatial tendency. Since the negative weights presented in our study are small or very small, it did not seem necessary to explore any further explanation of these signs.

The PLS-PM method revealed that small and medium companies constitute the main anthropogenic pressures responsible for the mortality of non-traumatic origin (R^2^=0.85), with a weight of w=10.74 and w=10.46, respectively. The stressor effect of small and medium companies in the non-traumatic death has a multifactorial origin. Industries typically have a large role in pollution due to smoke, water with chemicals, gas, landfills or disposable residues (with effects in short- and long-terms in reproduction and/or immunodepression). Besides, some studies have shown that urban/industrialized areas can induce changes in the ecosystem of the areas where they are located, such as warmer environmental conditions, seasonal changes, immunodepression in animals (as a result of the continuous high-stress level and presence of pollutants), higher density landfills, and presence of pests (e.g., rats that are a host of numerous pathogenic agents). Altogether, these factors may be responsible for leading to outbreaks of infectious and parasitic diseases [2,3,44,45].

A denser human presence ensures more probable detection of a dead animal, which in first appreciation may suggest a bias effect on the PLS-PM results and interpretations. It is worth noting, however, that the effect of human presence on the probability of detection equally affects all mortality causes and therefore will not significantly affect the PLS-PM results that focus strongly on detection of predominant mortality causes and weakly on detection of predominant mortality regions. Put another way, the spatial distribution of mortality causes may be influenced by population density, but the range of weights, path coefficients, and coefficients of determination in Figure-4a and b, which are the crux of PLS-PM results, will probably not. The same rationale holds for the potential effect of municipality size on the PLS-PM results. Some spatial distributions of pressures may be influenced by the municipality area (e.g., roads or small companies), but this circumstance will not necessarily affect the pressure weights in Figure-4a and b, because the pressure scores (e.g., km of roads or number of medium companies) in a specific municipality are proportional to the municipality area.

In our study sample, the number of animals with infectious (n=7) and parasitic diseases (n=7) was low. Some of the infectious diseases that were detected were leptospirosis (n=1) in the Order Carnivora; enteritis of unknown origin (n=1) in Order Rodentia; osteomyelitis (n=1) in Order Eulipotyphla; and myxomatosis (n=1) and rabbit hemorrhagic disease (n=1) in Order Lagomorpha. These cases could be related to contact with domestic and/or other wild animals’ carriers of these diseases, landfills, and immunodepression caused by stress. The parasitic diseases were mostly related to a high prevalence of ectoparasites in the Order Eulipotyphla and Lagomorpha. This could be related to the new climacteric conditions provided by the industries that allow the parasites to survive in winter potentiating parasitic infestations.

In the traumatic model, water reservoirs (w=–1.9), small companies (w=0.99), medium companies (w=0.81), and residential buildings (w=0.83) were the anthropogenic pressures mostly related to mortality of traumatic origin, with a corresponding path coefficient of 0.932. The great impact of water reservoirs in the mortality due to trauma in wild mammals can be related to many factors as well: the majority of those reservoirs are placed near agriculture and cattle raising areas facilitating the human-animal interaction. Farmers and shepherds still hunt and poach especially carnivores, to protect their livestock [46-49]. It was the case of a red fox (*Vulpes vulpes*) admitted with signs of gunshot and collected in an area with a proximity to some agriculture area with farm animals. Moreover, the water reservoirs proximity with urban or agrarian areas also promotes competition with pets (dogs and cats) and wild mammals run the risk of becoming preys of domestic animals [4,48,50,51]. It was the case of some red squirrels (*Sciurus vulgaris)*, bats (*Pipistrelus pipistrelus*, *Eptesicus serotinus*, and *Myotis myotis*), and hedgehogs (*Erinaceus europaeus)* admitted with lesions compatible with predation by cats and dogs (visible lesions caused by the teeth and claws) and that were collected near of urban areas. Respecting the other anthropogenic stressors identified by the PLS-PM model (small and medium companies and residential buildings concentration), the justification for its impact in the death of traumatic origin might be related with the infrastructures that favor the occurrence of accidents and with the intense movement of people and vehicles in those areas [52-55]. A considerable number of animals as red foxes (*V. vulpes*) (n=10), otters (*Lutra lutra)* (n=4), and hedgehogs (*E. europaeus)* (n=31) were admitted with traumatic lesions compatible with a collision with vehicles which could be related to its proximity with urban areas in search of food or new territory areas.

Residential buildings concentration was also related to animals’ death. This anthropogenic pressure might also constitute a risk associated with their infrastructures and human activity in its proximity. A particular example is the case of the bats that can be easily trapped inside the houses and suffer traumatic injuries [56-58]. It was possible to observe that many of the bats that were admitted in the WRC have been trapped inside houses and presented signs of collision with the walls and windows (as hematomas, fractures, and lacerations of the wing membrane and cerebral concussion). The high concentration of residential buildings and small and medium companies have been associated with a greater dispersal of infrastructures within a certain area leading habitat fragmentation and destruction [59]. Habitat fragmentation and destruction have been described by many authors as one of the main threats linked to the decline of wild populations [8,60-64]. The same phenomena may be present in northern Portugal; however, additional studies would be necessary.

We would like to point out that there may be a certain degree of bias in the relative percentages of the species and that it is related to the decision of the person who finds the animal to take it or not to the recovery center. Thus, some species may be more charismatic such as hedgehog, and therefore more susceptible to be delivery to WRC than others such as bats. Furthermore, small and medium-size animals (hedgehogs and rabbits) are easier to find and capture than larger and aggressive animals (e.g., wolves and wild pigs).

The results of PLS-PM regression established a relationship between anthropogenic pressures (intensification of urbanization and expansion of human population) and the mortality increase of wild mammals admitted to the WRC. Some species of more adaptable mammals (such as the red fox and hedgehogs) have been expanding their habitats to the increasingly urbanized areas. This phenomenon occurs not only by the loss of their natural habitats but also by the many benefits provided by these new biomes, such as easy access to food sources, shelter areas, and little presence of natural predators.

## Conclusion

Wild mammals are becoming more vulnerable to anthropogenic threats as collisions with vehicles, poisoning, or hunting. Due to the high rate of habitat destruction and fragmentation, the probability of direct contact between wild and human animals increases, with negative consequences for both, since they compete for the same natural resources (water reservoirs are a good example). The studies about interaction “wild mammals’ mortality versus anthropogenic pressures” are rather important since they provide the opportunity to identify species at risk, the geographic location of dominant anthropogenic pressures and also provide a realistic view of the ecosystem’s health. Therefore, the results of the present study may constitute a valuable tool to promote wild mammals’ conservation in northern Portugal and to help to educate people to value these species (in particular the predators) and to coexist with them.

## Authors’ Contributions

All authors contributed to the drafting of the manuscript. AG prepared the data, performed the data analysis and set up a manuscript proposal. FP and LSF were responsible for processing the data on the software. FQ, IP, and JP reviewed results and manuscript. VS and SL collected samples and animals’ follow-up. RC was responsible for the project supervision. All authors read and approved the final manuscript.
